# A Case of Frontotemporal Lobar Degeneration with Progressive Dysarthria

**DOI:** 10.1155/2006/320638

**Published:** 2006-07-17

**Authors:** Nami Ihori, Shigeo Araki, Kenji Ishihara, Mitsuru Kawamura

**Affiliations:** ^1^Department of RehabilitationKawasaki Cooperative HospitalKawasakiJapan; ^2^Department of NeurologyKawasaki Cooperative HospitalKawasakiJapan; ^3^Department of NeurologyShowa University School of MedicineTokyoJapan

**Keywords:** Frontotemporal lobar degeneration, progressive dysarthria, frontotemporal dementia, anterior operculum syndrome

## Abstract

We investigated the evolution of the neurological and neuropsychological characteristics in a right-handed woman who was 53-years-old at the onset and who showed personality changes and behavioral disorders accompanied by progressive dysarthria. She had hypernasality and a slow rate of speech with distorted consonants and vowels, which progressed as motor disturbances affecting her speech apparatus increased; finally, she became mute two years post onset. Her dysarthria due to bilateral voluntary facio-velo-linguo-pharyngeal paralysis accompanied with automatic-voluntary dissociation fit the description of anterior opercular syndrome. She showed personality changes and behavioral abnormalities from the initial stage of the disease, as is generally observed in frontotemporal degeneration (FTD), and her magnetic resonance image showed progressive atrophy in the frontotemporal lobes; thus, she was clinically diagnosed with FTLD. This patient’s symptoms suggest that FTLD, including bilateral anterior operculum degeneration, causes progressive pseudobulbar paretic dysarthria accompanied by clinical symptoms of FTD, which raises the possibility of a new clinical subtype in the FTLD spectrum.

## 1. Introduction

Since Mesulam [19,20] proposed primary progressive aphasia (PPA), several cases of degenerative dementia with isolated progressive deterioration in language function have been reported, and most of these might be classified as "Pick-lobar atrophy" complex or frontotemporal lobar degeneration (FTLD) [16,21]. In addition, there are case reports of degenerative dementia in which speech disturbances, as the initial symptom, progressed selectively [15]. Motor speech disorders are divided into two types, dysarthria and apraxia of speech. Different definitions of these terms by different authors have made it difficult to compare patients. We apply the definitions by Darley et al. [7]: dysarthria refers to speech disorders attributable to motor deficits, such as paralysis or ataxia of the speech organs, and apraxia of speech refers to those thought to be caused by deficits at a higher programming level of articulatory movements. The types of progressive speech disorders reported include apraxia of speech (e.g. [3,6,9]), apraxia of speech with non-fluent aphasia [14], apraxia of speech accompanied by late dysarthria (e.g. [3,11,26]), and dysarthria with language dysfunction (e.g. [12,25]). These include cases that might be clinically considered FTLD [9,11,14,26].

FTLD is a comprehensive concept proposed by the Manchester Group as a degenerative disease that causes non-Alzheimer's type dementia with primary lesions in the anterior regions of the brain; these have previously been called Pick's disease or frontal dementia [28]. The Manchester Group classified FTLD into three types, frontotemporal dementia (FTD), progressive aphasia

ISSN 0953-4180/06/$17.00 © 2006 – IOS Press and the authors. All rights reserved

(PA), and semantic dementia (SD), and described the pathological findings for each type. FTLD includes a few reports of patients with progressive dysarthria, not apraxia of speech.

Here, we report a case of progressive dysarthria as a symptom of anterior operculum syndrome. This patient showed personality and behavioral changes from the onset of the disease and her magnetic resonance image (MRI) showed progressive atrophy in the frontotemporal lobes; thus, she was diagnosed with frontotemporal lobar degeneration (FTLD). We investigated the neurological and neuropsychological characteristics of this patient. We report her clinical course and discuss the possibility of a new entity in the spectrum of FTLD.

## 2. Case

### 2.1. Case history

At onset, the patient was a 53-year-old right-handed woman with a high school education. Her medical history included hypothyroidism, which had been medicated for 20 years. She experienced difficulty in phonation in January 1997. At the same time, her behaviors became slower, and she gradually became lazy, not caring about housekeeping. In May 1997, she was referred to the neurology department of Kawasaki Cooperative Hospital with speech problems, and a detailed neurological and neuropsychological evaluation and speech therapy were started.

### 2.2. Neurological findings

No neurological problems were found, except for cognitive deficits and dysarthria. The main characteristic of dysarthria was reduced intensity and maximum duration of phonation. The vocal cords were symmetric and moved well under fiberscopic observation.

### 2.3. Phonation, resonance, and articulation

The intensity of phonation was decreased remarkably. Shortness in the duration of phonation (the maximum duration for [a:] was 3 seconds) and a breathy, harsh, and asthenic voice were observed without hypernasality. The range of movement of the tongue and lips was intact, but there was slight slowness during alternating movements. In oral diadochokinesis, the speed of articulation was mildly defective: 22 times for [pa], 26 for [ta], 24 for [ka], and 5 for [pataka] in 5 seconds each (Table 1). In spontaneous speech, monotonous intonation and a defective speech rhythm, including a shortage of long vowels and unnecessary prolongation of vowels, were observed without distortion in articulation. Singing was also disturbed, with a flat melody and abnormal rhythm.

### 2.4. Neuropsychological findings

Table 2 shows her neuropsychological test scores. The aphasia quotient of the Western Aphasia Battery (WAB) was 88.4 with almost intact auditory and visual comprehension, confrontation naming, repetition, and reading aloud. Economy of speech, i.e., aspontaneity of speaking and shortage of speech quantity, was observed with short and sometimes incomplete sentences. In spontaneous speech, paraphasias and syntactic problems were not observed. She wrote sentences with a few errors in kana characters.

Ideomotor, ideational, and buccofacial apraxia were not observed. She showed mild mental decline with attentional deficits. Apathy, loss of empathy for her grandchildren, limited facial expressions, indifference, and aspontaneity were observed (Table 3).

She underwent speech therapy as an outpatient once a week.

## 3. Evolution

### 3.1. November 1997 to August 1998

Tables 1, 2, and 3 show the evolution of her phonation, resonance, and articulation, neuropsychological test scores, and personality and behavioral changes at 6-month intervals. In November 1997, the intensity and maximum duration of phonation increased, probably due to the effectiveness of speech therapy; and her voice became strained instead of asthenic. In March 1998, hypernasality was noticed, as well as a complaint of the inability to gargle. In July 1998, the intensity and quality of phonation were unchanged, while the hypernasality had become more severe, and the maximum duration of phonation for [a:] decreased to 2 seconds. The rate and range of the voluntary alternating movements of the tongue (e.g., in-and-out and lateral movement to reach to the angles of the mouth) and lips (e.g., purse and retract the lips) became progressively defective. As motor disturbances affecting her speech apparatus increased, the distortion of the consonants and vowels with nasalization, reduced speech rate, and

Table 1
Evolution of phonation, resonance, and articulation

| | 1997.4 | 1997.11 | 1998.3 | 1998.7 |
|---|---|---|---|---|
| *Phonation* | | | | |
| Maximum duration [a:] | 3 sec | 6 sec | 6 sec | 2 sec |
| *Resonance* | | | | |
| Hypernasality | − | ± | + | ++ |
| *Oral diadochokinesis* (*times per second*) | | | | |
| [pa] | 4.4 | 1.7 | 2 | 1.5 |
| [ta] | 5.2 | 1.8 | 2 | 1.5 |
| [ka] | 4.8 | 1.7 | 1 | 1 |
| [pataka] | 1 | 0.5 | 0.5 | 0.5 |
| *Intelligibility** | 2 | 2 | 2–3 | 4–5 |

−: negative; ±: mild; +: moderate; ++: severe.
*: Intelligibility was scored using five levels: 1: can understand all that has been said; 2: cannot understand sometimes; 3: can understand when the content of speech is already known; 4: can understand sometimes; 5: cannot understand any words.

Table 2
Neuropsychological test scores

| | 1997.5 | 1997.11 | 1998.7 |
|---|---|---|---|
| WAB | | | |
| Spontaneous speech | 16 | 17 | 2 |
| Auditory comprehension | 9.8 | 8.6 | 8.2 |
| Repetition | 9.8 | 9.4 | 2.4 |
| Naming | 8.6 | 8.7 | 4.0 |
| Reading | 9.8 | 9.0 | 7.7 |
| Writing | 9.8 | 8.9 | 6.3 |
| AQ | 88.4 | 87.3 | 33.1 |
| Token test | | | |
| Auditory | 33/39, 158/167 | 31/39, 150/167 | 29/39, 150/167 |
| Visual | 30/39, 152/167 | 33/39, 155/167 | |
| 100 word naming test | 100/100 | 99/100 | |
| WAIS-R | | | |
| Verbal IQ | 68 | | 66 |
| Performance IQ | 62 | | 62 |
| Full-scale IQ | 61 | | 60 |
| MMSE | 27/30 | | 24/30 |
| Kohs block test IQ | 73 | 64 | 49 |
| RCPM | 21/36 | 22/36 | 23/36 |
| WMS-R | | | |
| Verbal memory | | 79 | 58 |
| Visual memory | | 97 | 77 |
| General memory | | 83 | 52 |
| Attention/Concentration | | 68 | 59 |
| Delayed recall | | 92 | 80 |

WAB: Western Aphasia Battery WAIS-R: Wechsler Adult Intelligent Scale Revised
MMSE: Mini mental state examination RCPM: Raven Colored Progressive Matrices
WMS-R: Wechsler Memory Scale Revised.

monotone became more marked in all speaking modalities including oral diadochokinesis, and her intelligibility became gradually defective (Table 1). As her articulatory problems progressed, the length of spontaneous speech became shorter, the content and grammatical structure of the sentences became simpler, and she often ceased speaking before completing a sentence. In July 1998 she lost all practical speech because of the progression of her articulatory problems, and she used writing for communication. Although she had some trouble in retrieving kana characters, she could communicate simple information by writing without difficulty. Singing was no longer possible.

Auditory comprehension remained mildly defective, and visual comprehension was well preserved until August 1998. The aphasia quotient in the WAB was 33.1, which was much lower than her previous scores, because of severe articulatory problems (Table 2). She scored 19 correct responses out of 20 in object naming in the WAB when writing, showing that her word-finding ability was preserved. However, the written description of a scenic picture in the WAB showed prominent perseveration with many errors in kana characters.

Ideomotor and ideational apraxia were not observed. On buccofacial praxis, she could follow verbal commands for elementary movements, such as protrusion and lateral movements of the tongue, whereas acts such as coughing and clicking the tongue were substituted with verbalization on both oral commands and imitation. However, parapraxis, such as tongue protrusion instead of coughing, was not observed.

Although her mental and memory functions did not show much deterioration on the neuropsychological test scores (Table 2), her personality and behavior changed remarkably (Table 3). Distractibility, apathy, loss of emotional expressions, aspontaneity gradually progressed, and in July 1998, disinhibition was so remarkable that she started responses before the examiner finished giving instructions for each task. In trail-making test A, she drew a line toward the numeral she looked at before attending to the order of the numerals.

She was admitted in June 1998 for one month to undergo a thorough neurological assessment. Ocular movements were full-range in automatic movements, but she could not pursue on command. Although the blink reflex was normal, and eye closure was possible while sleeping, she could not close her eyes intentionally on command. The palatal reflex was absent bilaterally, while the gag reflex was present. Jaw jerk was exaggerated, and a mask-like face and mild dysphagia were observed. Protrusion and lateral movements of the tongue were slow with restricted range; so were movements of the lips, jaw, and cheeks. Conversely, she could move her facio-linguo-pharyngeal muscles well when yawning and eating (automatic-voluntary dissociation). Neither atrophy nor fasciculation was observed in the tongue. Muscle strength and tone in the four limbs were normal. Sensory disturbances were not observed. She showed no involuntary movements. In the ward, she walked around peeping through the curtains around other patients at night (disinhibition).

### 3.2. *September 1998 to February 1999*

She stopped making herself up and went to the hospital without tidying herself in September 1998. At home, she repeatedly went upstairs and downstairs purposelessly. After the stairs were closed to her, because she fell several times, she repeatedly went to and from the bathroom. During meals, she continued putting food into her mouth until it spilled out. Although one of the authors continued her speech therapy once a week as an outpatient, the intensity and duration of phonation declined, and the asthenic quality became prominent. In writing, perseveration prevailed, and communication by writing was no longer possible. In November 1998, during a conversation with one of the authors, she repeatedly stood up and sat down without responding to the questions.

As her neurological condition had worsened, she was readmitted in December 1998. Bilateral pyramidal signs (brisk tendon reflexes, Babinski's sign, and spasticity in both lower limbs), extrapyramidal symptoms (postural instability, rigidity in the neck and upper limbs, and gait disturbances with initial freezing and small steps with acceleration), frontal lobe signs (palmomental reflex, snout reflex, pathological grasping, and stereotypical behaviors), and persistent automatic-voluntary dissociation in the facio-linguo-masticatory muscles were observed. On neuropsychological examination in February 1999, it was difficult for her to cooperate to accomplish the tasks. When she did pay attention to the examiner's instructions, she could follow simple oral commands such as, "Look at the window," and could point to objects named by the examiner. She did not utter any sound because of difficulty in phonation. She developed akinetic mutism about 2 years after the onset of symptoms, when her dysphagia had progressed to the point that she needed tube feeding. Electromyography performed in the right hand and leg showed neurogenic changes, suggesting anterior horn cell degeneration. Atrophy of the four extremities progressed, and she died of pneumonia about 7 and one-half years post onset.

## 4. Neuroradiological findings

On brain MRI in July 1998 (Fig. 1) and August 1999 (Fig. 2), progressive atrophy was observed in the frontal and temporal lobes. No abnormality was found on MRA. The HMPAO-SPECT performed in January 1998 showed hypoperfusion in the bilateral frontal and temporal cortexes and subcortical areas. The ECD-SPECT performed in June 1998 and August 1999 showed progressive hypoperfusion throughout the cerebral hemispheres.

Table 3
Changes in personality and behavioral abnormalities

| | 1997.5 | 1997.11 | 1998.3 | 1998.7 | 1998.12 |
|---|---|---|---|---|---|
| Attentional disturbances | + | + | + | + | ++ |
| Apathy | + | + | + | ++ | ++ |
| Loss of emotional expressions | ± | ± | + | ++ | ++ |
| Indifference to surroundings | ± | ± | ± | ++ | ++ |
| Aspontaneity | ± | ± | + | + | ++ |
| Distractibility | ± | + | + | ++ | ++ |
| Indifference to grooming | − | − | − | + | ++ |
| Stereotypy | − | − | − | + | ++ |
| Disinhibition | − | − | − | ++ | ++ |

−: negative; ±: mild; +: moderate; ++: severe.

## 5. Discussion

Our patient showed rapidly progressive dysarthria accompanied by progressive personality and behavioral abnormalities. The personality and behavioral changes in this patient started with mild indifference and aspontaneity, including reduced speech output, and then progressed to disinhibition and stereotypical behaviors, distractibility, perseveration, apathy, and indifference to personal grooming and hygiene. She finally showed akinetic mutism. These personality and behavioral changes are characteristic of FTD [16,28]. A neuroimaging study in this patient showed progressive frontotemporal atrophy without cerebral vascular disease. These clinical and neuroimaging findings strongly suggest that this patient had FTLD.

With regard to the speech characteristics in FTLD, FTD shows reduced speech output (economy of speech) without articulatory problems [24,28]. PA shows slow, effortful, and non-fluent speech with phonemic paraphasias and may involve apraxia of speech. The main characteristics of SD are semantic deficits with preserved phonological and phonetic aspects. Therefore, the speech disorders usually observed in FTLD are the result of aphasia or apraxia of speech; the exception is FTD with motor neuron disease (FTD-MND), in which progressive dysarthria due to bulbar and/or pseudobulbar palsy is present, often with wasting and fasciculation of the tongue [28]. Our patient showed dysphonia at the beginning of the disease, and then rapid progression of her articulatory problems with progressive motor deficits in the articulatory organs without atrophy or fasciculation of the tongue followed. Consequently, as the voluntary movements of the jaw, lips, tongue, and soft palate became defective, hypernasality, distortion of vowels and consonants, and slowness of speech in all situations increased. One and one-half years post onset, she lost all practical speech and used writing for communication. Therefore, the speech disturbance in this patient was considered to be attributable to dysarthria, not to aphasia or apraxia of speech.

The intact pharyngeal reflex, absence of a palatal reflex, exaggerated jaw jerk, and absence of atrophy or fasciculation in the tongue suggest that the dysarthria in this patient was not due to bulbar palsy, but to pseudobulbar palsy. In this patient, even in the progressive stage when it was getting difficult for her to open her mouth voluntarily, she could easily open it when eating or yawning (automatic-voluntary dissociation). In addition, pathological laughing and crying were absent. These features fit cortical pseudobulbar palsy or anterior operculum syndrome [1,2,4,10,22,23,31], the main symptoms of which are severe dysarthria of pseudobulbar type with bilateral central facio-linguo-velo-pharyngeo-masticatory paralysis, but with preserved automatic, involuntary, and emotional innervation (automatic-voluntary dissociation) [31].

Although anterior operculum syndrome develops most often after serial bilateral cerebrovascular accidents, there are a few reports of progressive anterior operculum syndrome as a result of neurodegenerative disease [2,18,22,27,30]. Case 1 in Arnould et al. [2] was a 50-year-old woman at onset, who showed dysphonia initially and then progressive dysarthria with hypernasality, until she finally lost her speech. The course of the progression of dysarthria in our patient seems to be most similar to the course in this patient. However, our patient is unique in that she also showed personality and behavioral changes from the initial stage of the disease. The case reported by Lang et al. [18], in which dysarthria was accompanied by progressive aphasia, showed hypoperfusion in the bilateral opercular regions on SPECT, predominantly on the left. Case 2 in Santens et al. [27] showed behavioral changes and aphasia during the course of the disease and demonstrated hypometabolism in the bilateral frontal and anterior temporal lobes on positron emission tomogra-

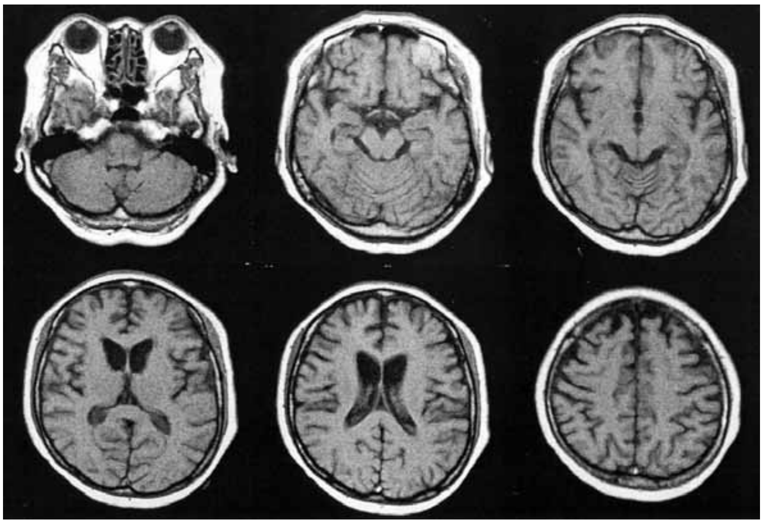

Fig. 1. Magnetic resonance imaging performed in July 1998 (one and one-half years after the initial stage of the disease). Atrophy in the frontotemporal regions is suggested.

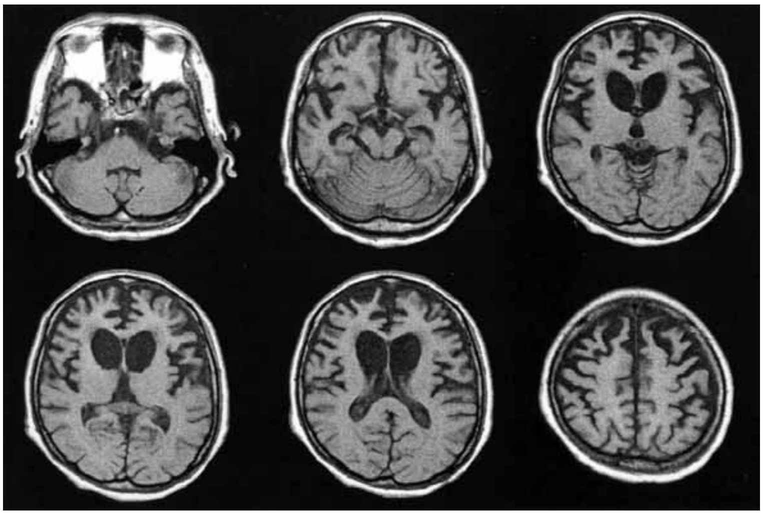

Fig. 2. Magnetic resonance imaging performed in August 1999 (two and one-half years after the initial stage of the disease). Compared with Fig. 1, progressive frontotemporal lobar atrophy is evident.

phy (PET). These cases could also be clinically considered FTLD. In case 1 by Nakajima et al. [22], the neuropathological findings suggested that she could be considered as FTLD [22,23].

By contrast, there are case reports with speech loss after the progression of pseudobulbar-type dysarthria attributable to degenerative disease other than progressive anterior operculum syndrome. In some of these reports, the clinical course of the speech disorders is described in detail, so that comparison with our case is possible [5,8,12,25]. The two patients reported by Fukusako et al. [12] showed dysarthria and apraxia of speech simultaneously, and aphasia appeared in the course of progression. The case of Sakajiri et al. [25] showed dysarthria with mild deficits in language and frontal-lobe function. In the cases reported by Caselli et al. [5], aphasia and dysarthria preceded motor neuron disease and progressed rapidly. The case of de Koning et al. [8] also showed mild deficits in frontal-lobe function. In all but the report by Fukusako et al. [12], all of the patients who underwent morphological brain imaging (CT or MRI) and functional brain imaging (SPECT or PET) showed atrophy or functional deficits in the bilateral frontal [8] or frontotemporal [5,

25] cortex, which suggests that these cases were also FTLD.

Therefore, it is possible that patients who demonstrate progressive pseudobulbar dysarthria in the course of evolution due to damage in the bilateral frontal or frontotemporal cortex may be included in FTLD. Especially when the patients have large lesions in the frontal and temporal lobes, including bilateral anterior opercular regions, as in our patient, they might show FTD with progressive dysarthria. These patients seem to form a new subgroup within the FTLD spectrum.

The most common pathology in FTLD includes FTD-MND, corticobasal degeneration (CBD), Pick disease, dementia lacking distinctive histology (DLDH), progressive supranuclear palsy (PSP) [13,17]. The pathological study in the cases with progressive anterior opercular syndrome shows DLDH [3,22]. Since our patient had the clinical findings which suggest MND, such as bilateral pyramidal tract signs, amyotrophy in all the limbs, and neurogenic changes in the electromyography in later stages, her pathological background seems to be closest to FTD-MND.

## Acknowledgments

M.K. was supported by CREST, and the Grant-in-Aid for Scientific Research on Priority Areas – System study on higher-order brain functions from MEXT (17022035). This study was also supported in part by a Showa University Grant-in-Aid for Innovative Collaborative Research Projects and a Special Research Grant-in-Aid for Development of Characteristic Education from MEXT.
